# A Natural Human Retrovirus Efficiently Complements Vectors Based on Murine Leukemia Virus

**DOI:** 10.1371/journal.pone.0003144

**Published:** 2008-09-04

**Authors:** Beihua Dong, Robert H. Silverman, Eugene S. Kandel

**Affiliations:** 1 Department of Cancer Biology, Lerner Research Institute, Cleveland Clinic, Cleveland, Ohio, United States of America; 2 Department of Molecular Genetics and Virology, Lerner Research Institute, Cleveland Clinic, Cleveland, Ohio, United States of America; Ordway Research Institute, United States of America

## Abstract

**Background:**

Murine Leukemia Virus (MLV) is a rodent gammaretrovirus that serves as the backbone for common gene delivery tools designed for experimental and therapeutic applications. Recently, an infectious gammaretrovirus designated XMRV has been identified in prostate cancer patients. The similarity between the MLV and XMRV genomes suggests a possibility that the two viruses may interact when present in the same cell.

**Methodology/Principal Findings:**

We tested the ability of XMRV to complement replication-deficient MLV vectors upon co-infection of cultured human cells. We observed that XMRV can facilitate the spread of these vectors from infected to uninfected cells. This functional complementation occurred without any gross rearrangements in the vector structure, and the co-infected cells produced as many as 10^4^ infectious vector particles per milliliter of culture medium.

**Conclusions/Significance:**

The possibility of encountering a helper virus when delivering MLV-based vectors to human cells in vitro and in vivo needs to be considered to ensure the safety of such procedures.

## Introduction

Development of vectors based on murine leukemia virus was a major step in the advent of experimental as well as therapeutic retroviral transduction[1]. With appropriate pseudotyping, MLV-based vectors could be used to transduce virtually any animal cell type, including those of mammalian, non-mammalian vertebrate and even invertebrate origin. Examples of the numerous applications include gene expression, insertional mutagenesis, marking of cells, as well as modeling the events of transcription and reverse transcription. Although replication-competent virus is occasionally used, most commonly the experiments are conducted with replication-deficient vectors. To this end, all or most of the protein-coding areas of the virus genome are removed and a “cargo” of interest is incorporated instead. The genomic transcript encompassing such a modified virus genome could be packaged in an infectious virion form if the missing protein functions are provided in trans, either transiently or from pre-integrated constructs in appropriately engineered packaging cells. While the virion may infect naïve cells, resulting in integration of the provirus in the DNA of the new host, the genes encoding the packaging proteins normally will not be transferred. Consequently, no further replication of the vector occurs and the virus will not spread beyond the progeny of the originally infected cell. This condition is heavily relied upon when the use of such vectors is considered. The situation would radically change if the viral proteins are expressed in the transduced cell, as might happen, for example, when the same cells are inhabited by a replication-competent MLV variant. This is a serious concern for the use of MLV in murine cells, where closely related retroviruses are sometimes found [2]. In addition, the proteins from related avian reticuloendotheliosis viruses (REV), such as spleen necrosis virus (SNV), could enable replication of MLV-based vectors [3]. On the other hand, the lack of reported natural replication-competent MLV variants in human population would make spontaneous complementation of replication-deficient vectors improbable. This was considered an additional safeguard for the use of these constructs, since a vector that enters human population during gene therapy or as a result of a laboratory accident is unlikely to spread.

Recently, a human retrovirus designated XMRV has been found in prostatic tissue samples from human prostate cancer patients [4], [5] and a closely-related virus has been found in at least one cultured human cell line (unpublished observation). XMRV was present in 8 (40%) of 20 patients homozygous for a reduced activity variant of RNase L, and in just 1 (1.5%) of 66 patients that harbored at least one copy of the wild type allele. XMRV displayed certain similarities with xenotropic strains of MLV, including three highly variable regions in the Env protein (SU or gp70) known to be important for species tropism, and was classified as a gammaretrovirus [5]. We decided to investigate whether the similarity between the two viruses may enable XMRV to complement a replicative defect of MLV-based vectors.

## Methods

Cells were cultured at 37°C in a 5% CO_2_ atmosphere in RPMI medium containing 10% FBS and supplemented with penicillin and streptomycin. Infections were carried out using filtered supernatants in the presence of polybrene as described elsewhere [6]. G418 selection was conducted at 0.5 mg/ml. The cells were fixed in methanol and visualized by staining with methylene blue (2% in methanol). DNA extraction was conducted using Blood and Cell Culture DNA Midi Kit from Qiagen, Inc. Southern blotting with a probe for the MLV packaging signal was described earlier [7]. The GFP-specific probe corresponded to the *Age I–Not I* fragment of pEGFP-N1 from Clontech. PCR was done with the earlier described MLV LTR-specific primers [8] and the primers within LNCE (sense primer: 5′-GGCCAGCAACTTATCTGTGT-3′; anti-sense primer: 5′-AGCTTGAGCTCGAGATCTGA-3′).

## Results

**Figure 1:**
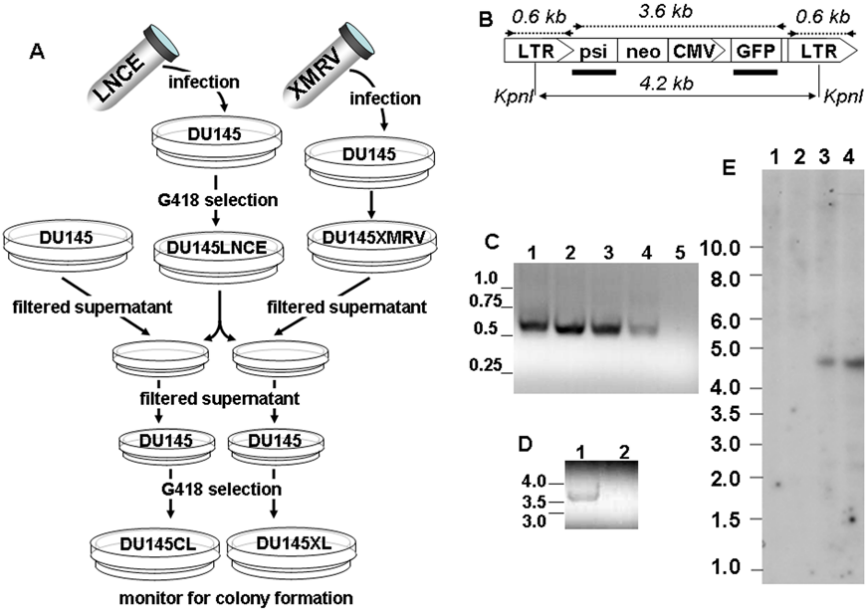
XMRV facilitates the transfer of MLV-based LNCE to naïve cells. A. The scheme of the experimental approach. DU145LNCE and DU145XMRV were generated by infecting DU145 cells with LNCE and XMRV respectively. The supernatant from DU145XMRV or fresh DU145 cells was applied onto DU145LNCE cultures. The treated cells were cultivated for additional week, and the presence of infection LNCE particles in the supernatant was tested by applying the latter to naïve DU145 cells (designated DU145XL or DU145CL respectively). The cells were selected in the presence of G418. No DU145CL cells survived the selection. B. Structure of LNCE[6]. LTR-MLV long terminal repeat; psi-MLV packaging signal; neo-neomycin resistance gene; CMV–cytomegalovirus promoter/enhancer region; GFP–the gene for enhanced green fluorescent protein. Positions of hybridization probes (thick lines) and PCR products (dotted lines) are shown below and above the diagram respectively. The predicted sizes of the PCR fragments and the distance between the two KpnI sites are indicated. C. Amplification of LNCE LTR from DU145XL cells. The expected product was obtained from the DNA of the pooled G418-resistant cells (lane 1) and several individually expanded clones (lanes 2–4), but not the original DU145 (lane 5). D. Amplification of an internal LNCE provirus fragment from DU145XL cells. The product was obtained from the DNA of DU145XL (lane 1), but not the naïve DU145 (lane 2). E. Detection of transmission of LNCE in the presence of XMRV by Southern blotting. Hybridization with the probe derived from the GFP fragment was performed on KpnI-digested DNA from DU145 (lane 1), DU145XMRV (lane 2), DU145LNCE (lane 3) and DU145XL (lane 4).

**Figure 2:**
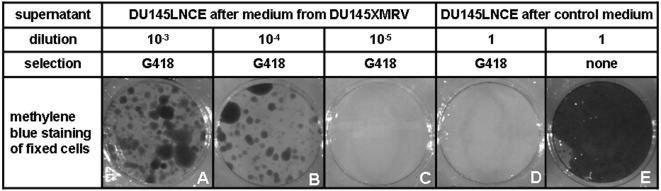
Determination of the titer of infectious LNCE in the presence of XMRV. The supernatant from DU145LNCE cells exposed to the medium from either DU145 (control medium) or DU145XMRV cells was applied in various dilutions to subconfluent cultures of naïve DU145 cells grown in 6-well plates. The treated cells were selected for G418 resistance and the surviving colonies were visualized by methylene blue staining. Neo-transducing particles were readily detectable in the supernatant of XMRV-exposed cells at 1,000-fold (panel A) and 10,000-fold (panel B), but not 100,000-fold (panel C) dilutions. The supernatant from DU145LNCE exposed to control medium was not toxic by itself (panel E), but it failed to transduce the resistance marker even when used without dilution (panel D).

In order to test the ability of XMRV to mobilize MLV-based vectors we devised an experimental scheme (Figure 1A) that utilized LNCE, a previously described MLV-based construct (Figure 1B) [6], which carries the genes for neomycin resistance and green fluorescent protein. DU145 human prostate carcinoma cells were stably transduced with LNCE, and a population of G418-resistant cells was obtained. As expected, this pool of cells, designated DU145LNCE, was nearly uniformly positive for GFP expression (data not shown). These cells were exposed to the filtered supernatant of DU145XMRV (DU145 cells that were previously infected with a replication-competent XMRV) and cultured for additional week to allow a potential helper virus to propagate. If XMRV could serve as a helper virus for LNCE, one may expect doubly-infected cells to produce infectious LNCE particles. To test this prediction, we collected the supernatant from XMRV-infected DU145LNCE cells, filtered it and applied to fresh DU145 cells. Two days later, the freshly infected cells were subjected to G418 selection. We observed massive formation of G418-resistant colonies, most of which showed detectable GFP expression by fluorescent microscopy (data not shown). No G418-resistant colonies were obtained in control experiment in which fresh DU145 cells were exposed to DU145LNCE supernatant in the absence of XMRV (e.g. Figure 2d). These observations suggest that XMRV can act as a helper virus for MLV-based vectors.

In principle, an alternative to direct functional complementation may be a recombination event, which would incorporate the elements necessary for XMRV replication into LNCE. However, co-expression of both marker genes in the newly infected cells argues that the process does not require gross rearrangements of LNCE. To further confirm the predicted structure of LNCE provirus in the newly infected cells we isolated DNA from the infected pool. Positive PCR signals were obtained using LNCE-specific primers (Figure 1C, D). Sequencing of the PCR-amplified material revealed that the fragment corresponding to the packaging signal of MLV is retained in the newly formed proviruses (data not shown). Furthermore, we analyzed KpnI-digested DNA from the infected cells by Southern blotting with the probes generated from GFP (Figure 1E) and MLV packaging signal (data not shown). The same 4.3 kb fragment appears to hybridize with both probes, as is expected for a full-length LNCE provirus. These observations confirm that in the presence of XMRV, LNCE genomes could be transferred to naïve cells without sustaining any major structural rearrangements.

In order to investigate how efficiently LNCE is mobilized by XMRV, we estimated the titer of LNCE that was produced by XMRV-infected DU145LNCE cells. The titer could not be accurately measured by a simple count of G418-resistant colonies: in the presence of a helper virus there is a possibility for LNCE to go through multiple rounds of replication, and thus more than one colony could originate from a single initial particle. To circumvent this problem, filtered supernatant conditioned by XMRV-infected DU145LNCE cells has been serially diluted, and the last dilution to contain the neo-transducing particles was determined. The estimated virus titer was approximately 10^4^ transducing particles per milliliter (Figure 2). This is comparable to what has been reported for similar MLV-based vectors produced in early-generation MLV-specific packaging cells. Of note, much higher virus yields obtained by modern techniques cannot be directly compared to our data since they are typically obtained either from individually selected high-producing clones or from cells harboring a high copy number of both the vector and the packaging constructs. Overall, we concluded that the ability of XMRV proteins to support propagation of replication-deficient MLV is comparable to that of the native MLV-derived factors.

## Discussion

Our observations provide additional experimental evidence in support of classifying XMRV as an MLV variant. Incorporation of unrelated RNAs in retroviral particles is possible, but inefficient. For example, SNV incorporates heterologous RNA at least 1000-fold less efficient than the RNA with proper packaging signals [9], [10]. Similarly, MLV is very efficient in packaging related mouse endogenous retroviruses, but not unrelated cellular RNAs [9]. Additional blocks at the level of reverse transcription and integration may further impede stable transduction of naïve cells with non-viral sequences, accounting for a nearly eight orders of magnitude difference between the replication efficiencies of constructs with or without specific retroviral cis-elements[10]. Various not mutually exclusive processes, such as aberrant splicing [11], or recombination between the viral and cellular sequences at the level of DNA[12], during transcription[13] and reverse transcription[14], [15] have been discussed as potential contributors to this process. However, neither of these mechanisms could explain the high frequency transduction of apparently intact LNCE in our experiments. Structure-based interactions in the absence of strong sequence homology have been alleged in cross-packaging of REV and MLV retroviruses [16]. However, the extended packaging signal of Moloney Murine Leukemia Virus, an MLV variant that served as the backbone for LNCE, displays about 70% homology with XMRV sequence. Therefore, the sequence-based recognition remains the most likely explanation for our observations.

The fact that a popular class of retroviral vector could readily become infectious in the presence of natural human virus has to be seriously considered whenever these constructs are being used, especially in a clinical setting. The most obvious concern is the possibility that a vector could accidentally encounter XMRV and would spread in human populations. In the case of gene therapy, the therapeutic construct might spread from the intended tissue target throughout the organism or even to additional individuals. An even more deleterious, albeit less probable, event might be an accidental escape of an experimental vector that harbors an oncogenic “cargo”. Modern MLV-based vectors have undergone extensive engineering to improve their performance in specialized tasks [1]. For example, mutations in the long terminal repeats have been used to improve expression, especially in embryonic cells[17]. An escape of such constructs would open a possibility for recombination with natural viruses, potentially increasing pathogenicity of the latter.

Another concern arises from the possibility to encounter XMRV in the cultures of human cells, where the virus might appear as the result of viremia of the original donor or due to laboratory contamination during establishment or further propagation of the cell lines. A pool of MLV-based constructs introduced into such cells will likely evolve over time and may spread to other cells, especially in co-culture experiments. In addition, in some instances live attenuated vaccines could potentially become contaminated with XMRV. Importantly, our preliminary observations suggest the presence of an XMRV-like retrovirus in a cultured cell line (unpublished observations).

It should be noted that a number of currently used vectors are so-called “self-inactivating”: after one round of replication their LTRs lose the promoter function. Promoter-deficiency is known to greatly diminish the risk of insertional mutagenesis [7], and it also decreases the risk of a vector-derived RNA being packaged in a virion in case the packaging proteins are introduced into the same cell. Unfortunately, this safeguard is unlikely to be full-proof: integration of the construct under the control of a cellular promoter might re-create a transcript that incorporates the vector genome, and RNAs with non-canonical placement of packaging signal could still be packaged [18].

Additional work is required to estimate the likelihood of XMRV-assisted propagation of MLV-based vectors in human population. So far, XMRV has been identified only in a limited number of humans, and the extent of its spread in human populations is unknown. Moreover, little is known about its tropism and the mode of infection. The association of XMRV infection with reduced function of RNase L may indicate that the virus could be sustained only in the individuals with decreased anti-viral responses [4], [5]. Moreover, co-packaging of XMRV and MLV genomes is yet to be documented, as is the recombination between the two viruses. Nevertheless, we believe that our observations warrant additional caution for the use of MLV-based vectors. In particular, they argue against the use of gene therapy vectors with functional LTRs; in favor of vector designs that minimize the likelihood of recombination with XMRV; and in favor of testing model human cell lines for the presence of MLV-complementing activity. In the meantime, it remains to be seen whether the differences between MLV and XMRV are enough to enable re-derivation of MLV-based vector systems that would lose the ability to be complemented by XMRV.
